# Comparison of Clean-Up Methods for Ochratoxin A on Wine, Beer, Roasted Coffee and Chili Commercialized in Italy

**DOI:** 10.3390/toxins5101827

**Published:** 2013-10-22

**Authors:** Ambra Prelle, Davide Spadaro, Aleksandra Denca, Angelo Garibaldi, Maria Lodovica Gullino

**Affiliations:** 1 Centre of Competence for the Innovation in the Agro-environmental Sector (AGROINNOVA), University of Turin, Grugliasco (TO) 10095, Italy; E-Mails: davide.spadaro@unito.it (D.S.); aleksandra.denca@unito.it (A.D.); angelo.garibaldi@unito.it (A.G.); marialodovica.gullino@unito.it (M.L.G.); 2 Department Agricultural, Forestry and Food Sciences (DISAFA), University of Turin, Grugliasco (TO) 10095, Italy

**Keywords:** mycotoxins, ochratoxins, occurrence, beer, wine, coffee, chili, HPLC

## Abstract

The most common technique used to detect ochratoxin A (OTA) in food matrices is based on extraction, clean-up, and chromatography detection. Different clean-up cartridges, such as immunoaffinity columns (IAC), molecular imprinting polymers (MIP), Mycosep™ 229, Mycospin™, and Oasis^®^ HLB (Hydrophilic Lipophilic balance) as solid phase extraction were tested to optimize the purification for red wine, beer, roasted coffee and chili. Recovery, reproducibility, reproducibility, limit of detection (LOD) and limit of quantification (LOQ) were calculated for each clean-up method. IAC demonstrated to be suitable for OTA analysis in wine and beer with recovery rate >90%, as well as Mycosep™ for wine and chili. On the contrary, MIP columns were the most appropriate to clean up coffee. A total of 120 samples (30 wines, 30 beers, 30 roasted coffee, 30 chili) marketed in Italy were analyzed, by applying the developed clean-up methods. Twenty-seven out of 120 samples analyzed (22.7%: two wines, five beers, eight coffees, and 12 chili) resulted positive to OTA. A higher incidence of OTA was found in chili (40.0%) more than wine (6.6%), beers (16.6%) and coffee (26.6%). Moreover, OTA concentration in chili was the highest detected, reaching 47.8 µg/kg. Furthermore, three samples (2.5%), two wines and one chili, exceeded the European threshold.

## 1. Introduction

**Figure 1:**
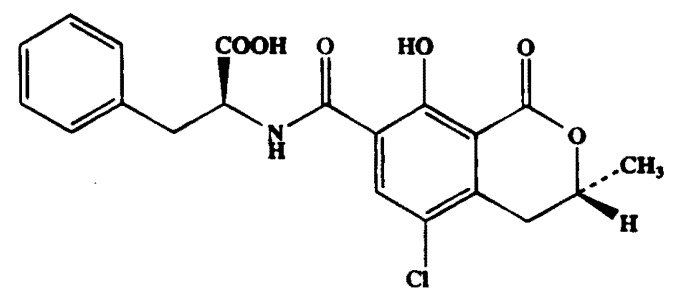
Chemical structure of OTA

Mycotoxins are secondary metabolites produced by fungal species growing on plant and on plant products with toxic effects to humans and animals [1]. Ochratoxin A (OTA; C_20_H_18_ClNO_6_) is an important mycotoxin produced by several species of *Aspergillus* (*A*. *ochraceus*, *A*. *melleus*, *A*. *nigri*, *A*. *carbonarius*) and *Penicillium* (*P*. *verrucosum*, *P*. *nordiucum*) [2] (Figure 1). As some *Penicillium* species can occur in cool climates and several *Aspergillus* species in tropical and subtropical regions, OTA can be found in a large variety of foods and beverages, such as cereals, cocoa, coffee, spices, wines, beers, and dried fruits [3,4,5,6,7,8,9,10,11,12]. Several studies have demonstrated that OTA is nephrotoxic and carcinogenic and its presence in food products poses serious threat to human and animal health [13,14,15]. Since 2003, OTA has been classified in the A2 group (possible carcinogen to human) by the International Agency for Research on Cancer [16]. Based on its potential health risk, the European Commission (EC) has established maximum limits for OTA content in different kinds of foods and beverages, including wine (2.0 µg/kg), roasted coffee (5.0 µg/kg), and spices (15 µg/kg) [17,18].

A large number of papers are published every year worldwide about the occurrence OTA in different foods and beverages. Wine has been the most investigated matrix, analyzed in different countries, such as Brazil [19], Spain [3,20], Italy [21,22], Chile [23], and Romania [24], followed by dried fruit and spices from Spain [25], Turkey [26], and Malaysia [12]; cereals [27,28,29,30,31,32,33], beers [3], and coffee [9,34,35,36,37].

OTA was identified and detected with several analytical methods, such as TLC [38], GC-MS [39], LC-MS [40], ICP (Inductively coupled plasma)-MS [41], LC-MS/MS [42], isotope dilution [43], aptamer [44,45,46], ELISA [47], and immunosensing methods [48], but the most common used technique is based on LC coupled with fluorimetric detector for high sensitive detection signal. Several methods have been reported for clean-up, which is necessary to remove matrix components that are usually present in the raw extract and can interfere by decreasing the sensitivity of the detection analysis. Different commercially available clean-up cartridges with several kinds of packing materials and sorbents, such as C-18, polymeric, and immunoaffinity have been applied to remove interferences and, in some cases, to concentrate the sample before chromatographic analysis. Clean-up columns can be divided in two categories: “pass through” and “capture the analyte” based on the clean-up interaction used. In the first strategy, the “pass through”, target compounds are not retained by sorbent, while all the interfering substances are strongly retained by the stationary phase. The other one, the most common strategy of SPE clean-up [49], use the opposite retention strategy, where analytes are strongly retained, while interfering compounds leave the cartridge during sample loading and washing. Different kinds of cartridges are commercially available for clean-up and pre-concentration, in particular immunoaffinity (IAC) and molecular imprinted polymers (MIPs) cartridges, composed respectively by anti-OTA antibodies and a three-dimension network specific for the target molecule. Both columns use the “capture the analyte” strategy. On the other hand, Mycosep™ and Mycospin™ cartridges, based on adsorption and ion-exchange process [42], are used only for clean-up and they exploit the “pass through” strategy. Despite the fact that IAC presents several problems, such as a rather high cost, cross reactivity and limited lifetime, on the basis of its high specificity, it is commonly used for OTA monitoring on several matrices [50]. Recent MIP columns, exhibiting several advantages, such as reusability, thermal stability, compatibility with all solvents, and longer shelf life, can be considered as an alternative to IAC. In fact many papers reported validation performances using MIP columns on different matrices, such as coffee [51], with recoveries from 90.6% to 99.4% for 1 to 50 µg/kg spiked levels; and recoveries > 92.1% on spiked cereals at 60 µg/kg [52]. Compared to the considerable use of IAC, Mycosep™ cartridges, despite the ease and speed of clean-up procedures, are less used. Due to their novelty, there are not, to our knowledge, any performance results about the use of Mycospin™ cartridge clean-up on OTA monitoring. To validate the methods, it is possible to use the European Reference Material (ERM) for specific mycotoxins and matrices. For OTA validation, ERM for red wine and roasted coffee are available, “ERM-BD476” and “ERM-BD475”, respectively.

Due to the high tendency to use IAC as clean-up procedures for mycotoxin analysis on different food without checking and validating the best procedure for each matrix, the aim of this work was to compare the IAC clean-up performance with other four different OTA selective cartridges commercially available (MIP, Mycosep™, Mycospin™, and HLB SPE). The study was carried out on four different matrices susceptible to OTA contamination: wine, beer, coffee, and chili. Recovery, reproducibility, repeatability were calculated for each method and matrix detecting OTA by HPLC coupled with fluorescence detector (FLD). The developed clean-up methods were applied to investigate OTA content in a total 120 samples of wine, beer, roasted coffee, and chilli, marketed in Italy. To the best of our knowledge this is the first report comparing five different clean-up methods for OTA detection (Figure 1).

## 2. Results and Discussion

The aim of this study was to compare and evaluate several clean-up methods used to quantify OTA content in four matrices. Sample clean-up and preconcentration steps are necessary to remove matrix components and enhance sensitivity before chromatographic analysis. Our approach was tested calculating recovery, repeatability, reproducibility, LOD, and LOQ, on five commercially available cartridges which differ on the binding phase and the clean-up strategy. To assess the effective recovery performance on OTA clean-up columns/cartridges, a preliminary experiment was carried out by loading standard solution at the lowest validation concentration tested (2 µg/L for wine and beer and 5 µg/L for coffee and chili). Standard solutions, simulating sample matrices, were prepared following extraction procedure used for wine, beer, coffee, and chili for each clean-up columns. All cartridges showed recoveries higher than 80%. 

To avoid OTA analytical signal loss caused by different ionic/neutral forms of the mycotoxin and consequently different absorption and emission bands, we used eluent solutions with 1% acetic acid to guarantee pH below 4 [52].

Based on the validation results obtained, we chose the clean-up method that led high recovery and repeatability and reproducibility, and used it to monitor OTA content in 120 samples of wines, beers, coffee, and chili.

### 2.1. Evaluation of Clean-Up on Wine

To determine the best clean-up cartridge method for recovering OTA in wine, two red wines produced in northern Italy were chosen, based on their low level of OTA contamination [53]. Table 1 shows the recovery rates obtained at different levels of concentration based on the selected cartridges. In agreement with OTA thresholds, established by EC for wine, the lowest level tested to validate the cartridge efficiency was 2.0 µg/L. Mycosep™ cartridges showed the lowest values for LOD and LOQ in comparison to the other clean-up methods considered. LOD values ranged from 0.12 µg/L for Mycosep™ to 2.02 µg/L for HLB SPE, while LOQ from 0.40 µg/L for Mycosep™ to 6.73 µg/L for HLB SPE. Recovery rate and repeatability (RSD) for Mycosep™ cartridges ranged from 96.75% to 102.68%, and from 2.46% to 24.48%, respectively. Reproducibility obtained ranged from 6.55% for Mycosep™ to 36.15% from SPE HLB. As we expected, RSDs value were higher for the lowest validation concentration for each clean up method, but, except for MIP column, it was below 25%. The loss of analyte at higher concentrations could be due to partial capacity saturation of cartridges by matrix component such as MIP for wine sample. Although RSD% obtained at 2 µg/L validation level exceeded the requirements of the Commission Regulation (RSD% ≤ 20), we chose this cartridge because in a single step we purified the matrix obtaining good recovery value comparable with those obtained by the more laborious IAC clean-up.

In general, an acceptable recovery rate (*R*% > 80) for most clean-up cartridges was obtained. On the other hand, the matrix interferences values calculated were different for “pass through” and “capture the analyte” cartridges, which induced respectively suppression and enhancement of the analytical signal. The good recovery and underestimation of analytical signal obtained by Mycosep™ column using the “pass through” strategy could be explained by the removal of wine compounds [54], such as polyphenols, which could interfere with the specific binding interactions limiting the column capacity of mycotoxin absorption.

### 2.2. Evaluation of Clean-Up on Beer

Because cereals are frequently contaminated by OTA and the mycotoxin is tolerant to the brewing process, OTA can be found in beers [55]. By considering the effect of different composition of lager, bitter and brown ale beer on mycotoxin recovery [56], we chose to work on lager beer, which is the most common beer. R, RSD, RSDs, LOD, and LOQ are listed in Table 2. The IAC cartridges provided a recovery ranging from 84.84% to 105.89%, and a RSDs from 9.09% to 25.41%, showing to be the best clean-up method for OTA in beer despite of RSD% for the lowest level of validation exceeded the requirements of the Commission Regulation (EC) No. 401/2006. RSDs values with for all clean-up method, ranging from 5.42 for HLB to 33.14 for MIP, resulted slightly higher than the repeatability. For the other purification methods, the recoveries never exceeded 62%. LOD value ranged from 0.08 µg/L for IAC to 0.29 µg/L for MIP, and LOQ from 0.26 µg/L for IAC to 0.97 µg/L for MIP. Differently from wine, where both IAC and column pass-through can be used, for beer, only IAC must be used to avoid losses and underestimation of OTA.

**Table 1:** Method validation parameters obtained from wine samples.

Clean-up method	Validation levels (µg/L)	Recovery (%) (*n* =6 )	RSD (%) (*n* = 6)	LOD (µg/L) (*n* = 6)	LOQ (µg/L) (*n* = 6)	RSDs (%) (*n* = 9)
IAC	2	84.03	19.22	0.14	0.48	20.15
	10	102.24	15.51			17.33
	20	93.07	10.53			11.96
MIP	2	80.46	27.01	0.14	0.48	36.15
	10	54.15	6.13			12.42
	20	70.31	9.67			10.41
Mycospin™	2	73.54	13.68	0.88	2.95	16.55
	10	77.76	21.65			15.11
	20	80.34	12.81			16.01
Mycosep™	2	96.75	24.48	0.12	0.40	20.63
	10	81.58	2.46			6.82
	20	102.68	3.69			6.55
HLB SPE	2	59.34	13.89	2.02	6.73	19.66
	10	83.49	18.56			19.38
	20	56.29	1.11			15.66

**Table 2:** Method validation parameters obtained from beer samples.

Clean-up method	Validation levels (µg/L)	Recovery (%) (*n* = 6)	RSD (%) (*n* = 6)	LOD (µg/L) (*n* = 6)	LOQ (µg/L) (*n* = 6)	RSDs (%) (*n* = 9)
IAC	2	84.84	25.41	0.08	0.26	21.44
	10	105.89	15.29			19.15
	20	99.2	9.09			14.33
MIP	2	62.99	30.25	0.29	0.97	33.14
	10	78.5	53.62			44.22
	20	75.98	16.64			23.10
Mycospin™	2	15.63	12.87	0.14	0.9	9.22
	10	53.15	9.22			10.03
	20	68.1	8.61			9.98
Mycosep™	2	67.65	27.58	0.27	0.48	31.69
	10	61.86	3.71			7.59
	20	66.35	14.1			13.97
HLB SPE	2	7.16	4.52	0.26	0.87	5.42
	10	27.15	8.53			15.96
	20	36.34	23.89			27.10

### 2.3. Evaluation of Clean-Up on Coffee

There is a wide literature documenting OTA content in coffee. Most studies focused on IAC as clean-up procedure [37,57], while only two studies used Mycosep™ [58] and MIP cartridges [51]. Based on the results obtained by recovery rate values, LOD, and LOQ, MIP columns resulted to be the best clean-up method able to purify the coffee matrix and reduce the matrix interference (Table 3). 

**Table 3:** Method validation parameters obtained from roasted coffee samples.

Clean-up method	Validation levels (µg/kg)	Recovery (%) (*n* = 6)	RSD (%) (*n* = 6)	LOD (µg/kg) (*n* = 6)	LOQ (µg/kg) (*n* = 6)	RSDs (%) (*n* = 9)
IAC	5	75.15	12.35	0.48	1.63	11.10
	10	83.68	2.16			6.23
	20	79.98	11.14			12.41
MIP	5	89.21	15.83	0.08	0.29	18.10
	10	82.31	1.99			3.15
	20	84.78	5.5			5.73
Mycospin™	5	33.61	33.81	3.03	10.12	30.62
	10	35.63	16.92			15.93
	20	38.91	25.93			16.22
Mycosep™	5	50.33	1.55	0.99	3.31	5.33
	10	49.98	12.8			15.11
	20	54.74	8.64			14.33
HLB SPE	5	71.51	26.34	0.22	0.74	30.94
	10	40.15	35.26			35.01
	20	43.91	20.14			21.95

*R* and RSD values obtained ranged respectively from 33.61 of Mycospin™ to 89.21 of MIP, and from 1.55 of Mycosep™ to 35.26 of HLB SPE. RSD and RSDs reported for the columns with specific binding interactions were below 16% obtaining higher recovery values than the other tested cartridge (ranging from 75.15% to 89.21%). Specific antibody-antigen interactions or molecular imprinted polymers are the most selective clean-up method on coffee. In agreement with the LOD and LOQ reported by Lee *et al*. on MIP clean-up on coffee [51], we obtained 0.08 and 0.29 µg/kg, respectively In light of the results obtained by Tozlovanu *et al*. [36], we could explain recoveries obtained with IAC clean-up. They explained that the antibodies identification was damaged by different type of interferences, such as the formation of open-ring OTA, due to the alkalinity of extraction solution (pH 7.9) and isomerisation of OTA during roasting, masking it from OTA-antibodies, while the presence of nonchlorinated analog OTB can interferes by cross-react with OTA-antibodies. 

### 2.4. Evaluation of Clean-Up on Chili

The complexity of chili matrix makes the extraction and analysis of mycotoxins more challenging, because most of the matrix components could interfere with the analytical signal. To our knowledge, all the studies about the OTA content in chili samples used IAC as a standard for clean-up [12,26,58,59]. LOD and LOQ obtained with all clean-up methods tested were higher than the previously tested matrices. In fact, LOD and LOQ values ranged from 0.30 µg/kg for Mycosep™ to 2.27 µg/kg for IAC, and from 1.00 µg/kg for Mycosep™ to 7.55 µg/kg for IAC. Contrarily to the published literature, IAC cartridges were not the best choice to reduce matrix interfering compounds, despite recovery rate ranged between 75.20% and 91.70%. Comparing with other clean-up methods, the highest values of LOD and LOQ, provided by OTA, could be explained by interaction of matrix component with the OTA-sorbent binding that led to an increase of baseline. Based on the validation results obtained, the Mycosep™ clean-up cartridge was chosen: LOD and LOQ values were 0.3 µg/kg and 1.00 µg/kg, respectively, while recovery rates and RSD ranged from 91.35% to 102.60% and from 1.37% to 8.12%, respectively (Table 4). 

**Table 4:** Method validation parameters obtained from chili samples.

Clean-up method	Validation levels (µg/kg)	Recovery (%) (*n* = 6)	RSD (%) (*n* = 6)	LOD (µg/kg) (*n* = 6)	LOQ (µg/kg) (*n* = 6)	RSDs (%) (*n* = 9)
IAC	2	75.20	5.20	2.27	7.55	7.17
	10	75.10	9.34			11.49
	20	91.70	2.23			7.13
MIP	2	83.81	31.58	1.77	5.93	34.52
	10	74.85	0.47			5.63
	20	81.00	1.95			6.18
Mycospin™	2	53.23	11.68	2.12	7.08	14.66
	10	65.14	18.66			21.46
	20	69.33	7.96			15.37
Mycosep™	2	91.35	8.12	0.30	1.00	9.17
	10	102.60	1.37			6.33
	20	96.60	2.85			4.82
HLB SPE	2	49.41	28.94	1.25	4.18	25.69
	10	57.88	35.80			33.15
	20	56.29	1.11			14.23

Based on the results obtained in this study, IAC column clean-up and the method applied demonstrated high efficacy to clean-up OTA. In particular, recovery values were never below 70%, RSD and RSDs values did not exceed 26% and 22%, respectively for all the matrices tested. Compared to IAC performance, MIP columns resulted more efficient, only for coffee clean-up, with recovery values higher than 80%, whilst for other matrices recovery values ranged from 54.15% (wine) to 83.81% (chili). Higher values of RSD and RSDs were obtained for the lowest validation values on MIP columns, highlighting the poor reproducibility on low OTA concentration. This effect was not present with the other concentrations tested. Clean-up performance of Mycosep™ cartridges resulted effective on wine and chili matrix, with recovery value above 80%; RSD and RSDs ranging from 1.37 to 24.48 and from 4.42 to 20.63, respectively. 

**Figure 2:**
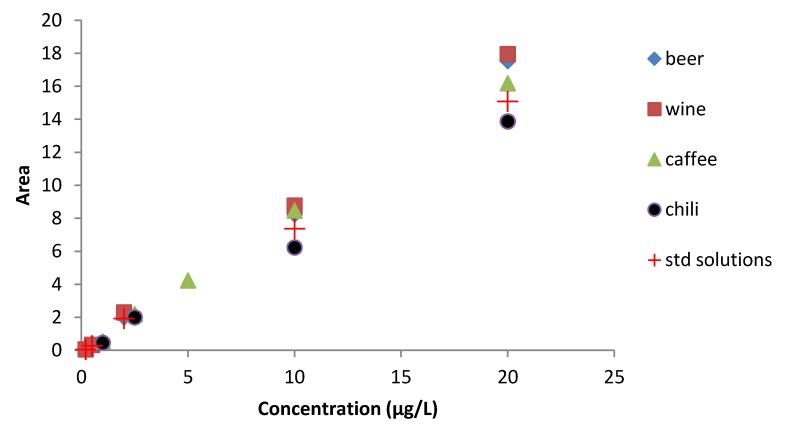
Matrix effect on calibration curve for wine, beer, coffee, and chili compared with the calibration curve obtained by the eluent solution.

To evaluate the matrix effect on the analytical signal we compared the calibration curves of the best clean-up of each matrix with the one in eluent solution (Figure 2). Clean-up method used resulted suitable to reduce matrix effect, in fact not many differences were highlighted. Positive signal enhancement of signal compared to the standard solutions occurred in beer, wine, and coffee matrices, while chili showed a slight decrease of analytical signal. Further studies will be necessary to explain matrix interactions in FLD. 

### 2.5. OTA Analysis in Commercial Food Samples

In Table 5 standard curves, calculated by linear regression of peak area against concentration values, were listed for each matrix previously treated with clean-up validated method. Linear regression value (*r*^2^) obtained, ranged from 0.9986 for wine and coffee, to 0.9997 for chili, assuring an adequate linearity. 

Based on the results obtained by the developed clean-up methods, we applied Mycosep™ cartridges to purified wine and chili samples, MIP for coffee and IAC for beer samples to determine OTA occurrence in different food product. In particular we analyzed 30 wines produced in Southern (10), Central (10), and Northern Italy (10); 30 European beers; 30 roasted coffee; and 30 chili, purchased in Italy (Table 6). In 27 out of 120 samples analyzed (22.5%), OTA was detected (two wines, five beers, eight roasted coffee, and 12 chili). In 16 positive samples, the contamination level was lower than 2.0 µg/Kg, in eight samples, ranged between 2.0 and 5.0 µg/Kg, in two samples, ranged between 5.0 and 15.0 µg/Kg, and in one sample the contamination was of 47.8 µg/Kg.

**Table 5:** Analytical performance of the clean-up method selected for the four matrices.

Matrix	Calibration range (µg/kg)	*r*^2^
Wine	0.2–20	0.9986
Beer	0.2–20	0.9910
Coffee	1–20	0.9986
Chili	1–20	0.9997

**Table 6:** Ochratoxin A (OTA) occurrence in wine, beer, coffee, and chili products marketed in Italy.

Matrix	Positive/Total (%)	Average contamination ± SD in positive samples (µg/kg)	Distribution of samples (µg/kg)			
			LOD–2.0	2.0–5.0	5.0–15.0	>15.0
Wine	2/30 (6.7%)	2.34 ± 0.35	-	2	-	-
Beer	5/30 (16.7%)	0.35 ± 0.06	5	-	-	-
Coffee	8/30 (26.7%)	1.03 ± 0.17	7	1	-	-
Chili	12/30 (40.0%)	8.45 ± 1.73	4	5	2	1

In agreement with the literature showing a higher contamination of red wines produced in Southern Europe compared to Northern regions, due to favourable climate conditions for fungal growth [53], OTA was only detected in two samples from Southern Italy. OTA contamination was slightly above the EU threshold. Remiro *et al*. [60] published a recent OTA monitoring on different red wines from Mediterranean countries and found a high contamination incidence (99% of the samples, with an average concentration of 0.054 µg/L). 

Regarding the OTA content in beer, 5 out of 30 beers analyzed contained OTA below 2.0 µg/L. Currently EU Commission has not yet fixed the maximum admitted level of OTA concentration in beer. Recent monitoring about OTA content in beers were performed by Skarkova *et al*. [57], Cao *et al*. [42], and Al-Taher *et al*. [43], which analyzed respectively 24, 10, and 76 samples. OTA was only detected by Skarkova *et al*. in 22 out of 24 samples, with concentration levels below 0.2 µg/L.

OTA was detected in 8 out of 30 samples of roasted coffee, where no one sample exceeded the maximum level established by EU. In particular, OTA concentration in seven out of eight positive samples was lower than 2.0 µg/kg, and only one sample overstepped 2.0 µg/kg. Data obtained are in agreement with the findings of other recent papers on OTA. Drunday *et al*. [36] analyzed 22 samples of coffee including roasted coffee, and reported 17 samples contaminated by OTA with average concentration lower than 2.0 µg/kg, while Tozlovanu *et al*. [37] reported that 81% coffee had OTA level below European limits and only three samples exceed 5 µg/Kg.

Chili products showed the highest incidence of contamination, compared to the other matrices, with 12 out of 30 samples positive: four samples were contaminated at levels below 2.0 µg/kg, five samples between 2.0 and 5.0 µg/kg, and two between 5.0 and 15.0 µg/kg. One sample was contaminated by more 15.0 µg/kg OTA, exceeding the threshold established by the EU Commission. Compared to other countries, such as Pakistan [58], where OTA was detected in 23 chili samples out of 63, with an average concentration of 22.15 µg/kg, the situation of chili commercialised in Italy guarantees a higher level of food safety and consumer protection.

## 3. Experimental Section

### 3.1. Chemicals and Reagents

All solvents used to extract, to activate, to condition, and to elute OTA by clean-up columns and used as eluent during HPLC determination were HPLC and LC-MS grade. Methanol, acetonitrile (ACN), *N*-hexane, acid acetic and hydrochloric acid 30% were purchased from Sigma-Aldrich (St Louis, MO, USA). Eluents were degassed daily for five minutes and filtered through mixed cellulose ester 0.22 μm-filters (Advantec MFS, Inc., Pleasanton, CA, USA) before use. Sodium chloride, sodium hydrogen carbonate, and polyethyleneglycol (PEG) were obtained from VWR (VWR International, Milano, Italy). Solid-phase extraction (SPE) was performed using a 24-position SPE vacuum manifold from Agilent Technologies (Santa Clara, CA, USA). Clean-up column tested were: Mycosep™ 229 and Mycospin™ columns, supplied by Romer Labs. (Union, MO, USA), Oasis^®^ HLB from Waters (Milford, MA, USA) with 500 mg of stationary phase, OtaClean select immunoaffinity columns from LCTech (Dorfen, Germany), and AffiniMIP for OTA from Polyintell (Val-De-Reuill, France). All samples were filtered through regenerated cellulose (RC) filters 0.45 μm (Advantec MFS, Inc., Pleasanton, CA, USA). OTA standards at 100 µg/mL in ACN were purchased from Sigma-Aldrich and diluted with mixture H_2_O:ACN:CH_3_COOH (90:9:1) to prepare individual standard stock solutions to obtain calibration curves, matrix interference value, recovery, for each extraction and clean-up method. All standard solutions were stored in the dark at −20 °C. Ultra-pure water was obtained from Maina system (G.Maina, Italy). 

### 3.2. Sample Preparation

Four different matrices, two liquids (red wine and beer) and two solids (roasted powder coffee and chili), were chosen to optimize and validate the analytical clean-up methods. OTA content was monitored in 30 samples of each matrix purchased in Italian supermarkets by using the optimized clean-up method. In the first part of this study, concerning the optimization and validation of clean-up method, two samples for each matrix, which confirmed to be OTA free, were used. The absence of OTA, was confirmed by analysis through LC-MS/MS as follows: one aliquot of the sample was analyzed as such, whilst other aliquots were spiked with a known concentration of mycotoxin standard, for each extraction method tested. Food samples were prepared, extracted, analyzed, and compared with calibration curves obtained by OTA standard solutions. 

To avoid the OTA convertion to an open-ring molecule at pH above 8 with consequent loss of recognition by OTA-antibodies pH of diluted extraction solutions were measured before IAC clean-up step ensuring a neutral pH value. 

Due to the different materials and interaction mechanisms of the columns/cartridge chosen, we decided followed the extraction protocols for each matrix, as suggested by column/cartridge manufacturer product, in particular, Romer laboratory for Mycosep™ and Mycospin™, Polyntell for MIP columns and LCTech for IAC.

For the extraction and clean-up validation on wine, two commercial red wines were chosen, transferred in amber glass bottles and stored in the fridge. IAC clean-up experiments were performed following the validated method described by Spadaro *et al*. [53], whose results were comparable to the manufacturer’s method (supplementary Table 1). For the MIP columns 10 mL of wine was diluted with 10 mL of HCl 0.1N. According to extraction protocol of Romer labs, for Mycosep™ and Mycospin™ cartridges, 25 mL of wine was diluted with 100 mL of a mixture of ACN and water (84:16 *v*/*v*), and 1 mL of acetic acid was added for extraction with Mycospin™ cartridge. The same extraction mixture used with Mycosep™ columns was also applied for Oasis^®^ column.

Similar protocols applied for wine samples was used to determine the best clean-up procedure for beer. Two lager beers were ultrasonicated for 20 min to eliminate most of the inner gas, before clean-up procedures. 

Concerning the validation on roasted powder coffee, for IAC and HLB SPE method 10 g aliquot of the powder sample was extracted with 100 mL of mixture (50:50; NaHCO_3_ 3%: methanol) solution for 30 minutes; while for MIP column 10 g aliquot of the powder sample was extracted with 100 mL of NaHCO_3_ 3%: solution. For Mycosep™ clean-up columns 25 g of sample was weighted in a 250-mL Pyrex flask and extracted with ACN:H_2_O 84:16 (*w*/*w*) mixture for 30 min. For Mycospin™ purification columns, the same extraction protocol was used, by adding 0.2 mL of acetic acid to the extraction solution.

Chili samples were pulverized using a food processor until homogeneous and extracted with HLB SPE, MIP, and Mycospin™ applying the same extraction protocol used for MIP coffee sample extraction. Instead for Mycosep™ 4 g of sample were put in a 50 mL centrifuge tube with 20 mL of extraction solution, methanol:water (80:20; *w*/*w*), and left for five minutes in ultrasonic apparatus at 25 °C. For IAC 2 g of NaCl was added to 20 g of sample and extracted for five minutes with 100 mL of mixture (80:20; methanol:H_2_O) and 50 ml of *N*-hexane. To accelerate phase separation the extract was centrifuged at 2000 rpm for five minutes. The water phase was taken and diluted with PBS solution. To separate the solid sample from the extraction solutions, before the purification step, centrifugation for 15 min at 6000 rpm, and filtration through a Whatman PVDF 0.45 µm syringe filter were performed for all extracted samples. All samples were stored in the dark at 4 °C with low relative humidity before analysis.

### 3.3. Clean-Up Steps

IAC, MIP and HLB SPE were carried out with a 24-position SPE vacuum manifold from Supelco. Despite the different interactions between stationary phase and analyte, all the cartridges follow the same procedure: activation, sample loading, washing, and analyte elution. 

Before IAC clean-up step all extraction sample solutions were diluted twenty times in PBS to reduce the pH below 8. Ten milliliters of diluted sample solution, for all matrices, were loaded into IAC column and, then, 5 mL of washing solution (2.5% NaCl and 0.25% NaHCO_3_) and 5 mL of ultrapure water were added into the column. Before elution with 3 mL of methanol into an amber glass vial, the column was air dried.

The used MIP procedure was, also, similar for each kind of matrix, except for the loading volume (2 mL for wine and beer, and 4 mL for chili and coffee). The MIP cartridge was previously conditioned with 4 mL of ACN and equilibrated with 4 mL of water at a flow rate of 1 drop/s. After sample loading, the cartridge was washed with 7 mL of HCl 0.1 N/CH_3_CN (60:40, *v*/*v*). At last, OTA was eluted with 2 mL of methanol (2% acetic acid) and collected in an amber glass vial.

The HLB SPE cartridges were activated with 5 mL methanol, following the manufacturer’s procedure, and conditioned with 5 mL of HCl 0.1 N solution. 5 mL of diluted sample was loaded into the cartridges and, before elution, sorbents were washed with 2 mL ultrapure water followed by air drying. Three milliliters of methanol was used as elution solvent.

To clean-up with Mycosep™, 70 µL of acetic acid were added to 7 mL of centrifuged and filtrated extract and pressed though the cartridge. Four milliliters of cleaned extract was evaporated until dry. Following the Mycospin™ manufacturer’s procedure, 1 mL of sample extract was placed into the column and vortexed for one minute. After vortexing, columns were put into 2 mL centrifuge tube and were centrifuged for three minutes at 10,000 rpm. Two hundred microliters of purified extract was evaporated.

All elutes were evaporated at 50 °C and the residues were dissolved in LC mobile phase and vortexed until dissolved and homogenized. Before injection in HPLC apparatus, all samples were filtered through a 0.45 RC syringe filter. All samples were analyzed in triplicate.

### 3.4. HPLC and LC-MS/MS Conditions

OTA was detected with a HPLC apparatus 1100 series Agilent Technologies equipped with G1311 quaternary pump, G1379 degasser, G1313A autosampler, G1316A column thermostat and G1321A FLD—Fluorescence Detector. The mobile phase consisted in an isocratic mixture of ACN:water:acetic acid (49:49:2) for 15 min. Each sample (30 µL) was injected into the analytical column Synergi 4 µ Hydro-RP (250 mm × 4.6 mm, Phenomenex, Torrance, CA, USA) and detected using 333 and 460 nm as wavelengths for excitation and emission, respectively.

Liquid chromatography coupled with mass spectrometry was used to confirm OTA absence in samples used for clean-up validation method. 1260 Agilent Technologies consisting of binary LC pump and a vacuum degasser; connected with a Varian autosampler Model 410 Prostar (Hansen Way, CA, USA) equipped a 100 µL loop was used as liquid chromatograph and was coupled to a triple quadrupole mass spectrometer Varian 310-MS. The mobile phase consisted of an isocratic mixture of ACN:water:acetic acid (50:50:0.1) for 15 min. Each sample (10 µL) was injected into the analytical column Synergi Fusion RP (100 mm × 4.6 mm, Phenomenex, Torrance, CA, USA).

Varian 310-MS was operated in positive electrospray ionization mode (ESI^+^). The ionization source conditions were: needle voltage of 2.5 kV, capillary voltage of 60–77 V, source temperature of 50 °C, desolvation temperature of 350 °C, cone gas flow rate of 50 psi, desolvation gas flow rate of 50 psi with nitrogen. Multiple reaction monitoring (MRM) mode of operation was used. The most intense daughter ions, resulting from collision-induced dissociation with argon, were used to detect and quantify OTA content. The argon pressure was set at 1.8 psi. Two daughter ions detected were: *m*/*z* 404→358 at 18 eV of collision energy (CE) and *m*/*z* 404→239 at 30 eV CE.

### 3.5. Validation

The validation of the optimized clean-up method includes possible matrix interferences (MI), apparent recovery rate (R), repeatability (RSD), reproducibility (RSDs), limits of detection (LOD), and quantification (LOQ). Following the guidelines of Commission Decision 2002/657/EC [61], these parameters were validated. *R*(%) is the ratio of mass released, in particular the percentage of OTA released from cartridge compared to the mass originally loaded. To evaluate the *R* and RSD, a total of two blank samples independently for each matrix were spiked with OTA standards to achieve three different concentrations, prior to extraction. The spiking levels for each matrix were chosen according to the European threshold levels, 2, 10, and 20 µg/L for wine and beer; 5, 10, and 20 µg/kg for coffee and chili. RSDs values were obtained analysing one blank sample spiked at three levels of validation in three different days by the same operator.

The amount of spiked solvent in “blank sample matrix” did not exceed 0.06% in volume. The results were compared with OTA standards prepared in the elution solvent at the same concentrations. The spiked samples of coffee and chili were left at room temperature for an hour for solvent evaporation. Linear regression analyses were obtained for each matrix. Five-point calibration curve was plotted at different concentrations (0.2, 0.5, 2, 10, and, 20 µg/L for wine and beer and 1, 2.5, 5, 10, and, 20 µg/kg for coffee and chili matrices). Each point was repeated in triplicate. The limits of detection (LOD) and quantification (LOQ) of each method were assessed. LOD was defined as three times the electronic baseline noise and LOQ as ten times the level of the baseline noise. The baseline noise was obtained with a blank sample for each matrix processed following the tested procedures. The recovery (*R*) was calculated following the formulas proposed by Matuszewski *et al*. [62]:

*R*(%) = C/B × 100 (1)

where B is the average concentration in the blank sample matrix spiked after clean-up step and C is the average concentration in the sample extract spiked before extraction; all spiked at the same concentration. The data obtained from these experiments conducted in single and three days were used to study the intraday and inter-day precision by calculating RSD(%) and RSDs (%). 

## 4. Conclusions

In conclusion, we compared different clean-up techniques commercially available regarding their recoveries, repeatability, matrix interferences and LOD and LOQ for OTA detection in different food matrices, such as wine, beer, roasted coffee, and chili. The two categories of clean-up strategies tested, based on purification interaction used, “pass through” and “capture the analyte” demonstrated different ability to remove interference or capture the analyte depending on matrix. IAC columns revealed high efficacy for OTA clean-up in each matrix, with recovery values never below 70%. Compared to IAC performance, MIP columns resulted more efficacy only for coffee clean-up with recovery value >80%, whilst for other matrices recovery values ranged from 54.15% for wine matrix to 83.81% for chili matrix. Several studies will be necessary to understand which matrix compounds and method conditions interfere with specific bond-capacity of MIP.

Despite the different strategy of clean-up used by Mycosep™ cartridge which does not permit sample enrichment, validation results demonstrated high efficacy on wine and chili matrix with recovery value above 80%. 

In addition, the chosen clean up procedures were used to monitor the situation of red wines, beers, roasted coffee, and chili commercialized in Italy. Twenty-seven out of 120 samples analyzed resulted positive to OTA, but just three samples (2.5%), two wines and one chili, exceeded the European threshold. Due to the limited number of samples analyzed, the monitoring should be enlarged to include more matrices. Anyway, a certain level of attention should be kept, because a limited number of samples are not legally marketable on the European market. This paper highlights the importance of choosing the suitable clean-up column/cartridge to be used when validating a mycotoxin detection method for a new matrix. 

## Supplementary Files

Supplementary File 1 Supplementary Table 1 (PDF, 7 KB)
