# Supplementary material for: Comparison of Clean-Up Methods for Ochratoxin A on Wine, Beer, Roasted Coffee and Chili Commercialized in Italy
Source: Toxins (Basel). 2013 Oct 22;5(10):1827–44. doi: 10.3390/toxins5101827 (PMC3813914; doi:10.3390/toxins5101827)
Supplement: Supplementary File 1 — Supplementary Table 1 (PDF, 7 KB) [file toxins-05-01827-s001.pdf]

Supplementary Table 1. Comparison between Spadaro's and manufacture's methods in wine matrix.

**Clean-up method**	**Validation levels (μg/L)**	**Recovery (%) (*n* = 3)**	**RSD (%) (*n* = 3)**	**LOD (μg/L) (*n* = 3)**	**LOQ (μg/L) (*n* = 3)**
Method of Spadaro *et al.*	2	84.03	19.22	0.14	0.48
	10	102.24	15.51		
	20	93.07	10.53		
Method of manufacturer	2	82.31	17.84	0.12	0.41
	10	99.15	12.26		
	20	93.96	13.95		
